# Synthesis and Antifungal Activities of Some Novel Pyrimidine Derivatives

**DOI:** 10.3390/molecules16075618

**Published:** 2011-06-30

**Authors:** Li Sun, Jie Wu, Lingzi Zhang, Min Luo, Dequn Sun

**Affiliations:** Marine College, Shandong University, Weihai Wenhua West Road, No.180, Weihai 264209, China

**Keywords:** pyrimidine derivatives, synthesis, antifungal activity, SAR

## Abstract

Three series of new pyrimidine derivatives were synthesized and their antifungal activities were evaluated *in vitro* against fourteen phytopathogenic fungi. The results indicated that most of the synthesized compounds possessed fungicidal activities and some of them are more potent than the control fungicides. Preliminary SAR was also discussed.

## 1. Introduction

**Figure 1:**
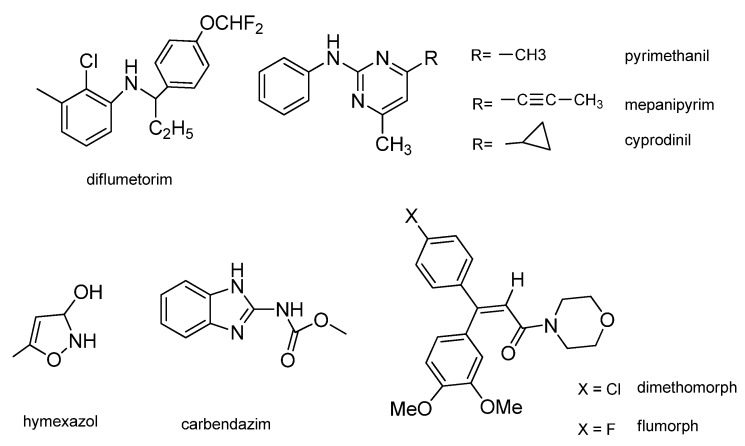
Chemical structures of several commercial fungicides.

**Scheme 1:**
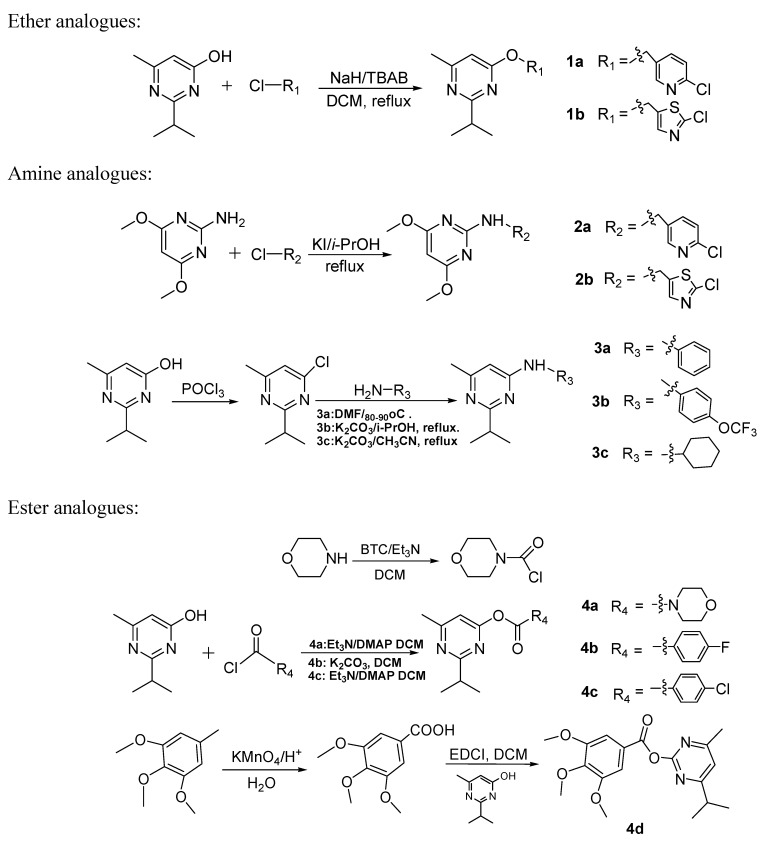
Synthetic routes to the three series of pyrimidine derivatives.

Phytopathogenic fungi that easily infect many crops are hard to control and risk resistance to the widely used commercial fungicides [1], therefore, there is a continuous need for new classes of antifungal agents. Natural compounds containing pyrimidine skeletons, such as vitamin B_1_ [2,3], and nucleotide bases [4] play an important role in life science studies. Pyrimidine derivatives have attracted great interest due to their diverse biological activities. For example, Rashad [5] synthesized a series of 4-hydrazinopyrimidine derivatives with *in vitro* antimicrobial activity. Rotili [6] reported a novel series of diarylpyrimidine and dihydrobenzyloxopyrimidine hybrids endowed with high and wide-spectrum anti-HIV-1 activity both in cellular and enzyme assays. Different classes of pyrimidine derivatives were synthesized and screened for antitumor activity to give candidates in drug discovery [7,8]. Pyrimidine derivatives have also occupied a prominent place in the field of agrochemicals because of their significant properties as fungicides in agriculture. To date, some important commercial pyrimidine fungicides, such as azoxystrobin [9,10], cyprodinil [11], pyrimethanil [12], and diflumetorim [13] (Figure 1) have been used in agriculture. Encouraged by the numerous pharmacological activities of pyrimidine derivatives, we were prompted to develop some novel pyrimidine fungicides. Three series of new pyrimidine derivatives **1–4** were designed and synthesized. The synthetic routes are shown in Scheme 1. All of the new compounds were evaluated *in vitro* for their antifungal activities against fourteen phytopathogenic fungi and the results of preliminary bioassays were compared with those of some commercial agricultural fungicides: flumorph, dimethomorph, carbendazim, hymexazole and pyrimethanil (Figure 1), which were currently used in the field in China. The results indicated that most of the pyrimidine derivatives possessed certain fungicidal activities, and the preliminary SAR of these compounds was investigated.

## 2. Results and Discussion

### 2.1. Synthesis

Compounds **1a** and **1b** were prepared by an improved method using NaH and TBAB in freshly distilled DCM since they couldn’t be obtained according to the method reported in the literature [14]. Compounds **2a** and **2b** were obtained according to the similar method in literature [15] in the presence of KI. Using POCl_3_ as chlorination reagent [16], the intermediate 4-chloro-2-isopropyl-6-methyl-pyrimidine was synthesized and used for the next substitution directly without purification in a small-scale synthesis. Reacting 4-chloro-2-isopropyl-6-methylpyrimidine with the corresponding aminesin different solvents afforded **3a** [17], **3b** [18] and **3c** [19], however, it was not necessary to use K_2_CO_3_ in the preparation of **3a**. In the preparation of compound **4a**, the 4-morpholinecarbonyl chloride must be prepared freshly before use. Et_3_N and K_2_CO_3_ were employed in preparation of **4b** and **4c** respectively, since there were more by-products when Et_3_N was used in preparing **4b** while few impurities were noted while synthesizing **4c**. After the 3,4,5-trimethoxybenzoic acid was ready, the condensation with 6-hydroxy-2-isopropyl-4-methylpyrimidine using EDCI as coupling reagent gave **4c**. Most of the compounds were prepared is yields of over 50%.

### 2.2. Antifungal Bioassay: Inhibitory Effects on Phytopathogenic Fungi

The fourteen phytopathogenic fungi chosen included *Alternaria kukuchiana* (AK), *Alternaria mali* (AM), *Alternaria solani* (AS), *Botrytis cinerea* (BC), *Bipolaris maydi* (BM), *Cercospora arachidicola* (CA), *Gibberella zeae* (GZ), *Gibberella fujikuroi* (GF), *Macrophoma kuwatsukai* (MK), *Phytophthora infestans* (Mont) de Bary (PI), *Rhizoctonia solani* (RS), *Rhizoctonia cerealis* (RC), *Sclerotinia sclerotiorum* (SS), *Thanatephorus cucumeris* (Frank), Domk (TC). All the fungi are typical and often occur in the Chinese agro-ecosystem. The antifungal activities of the eleven pyrimidine derivatives were investigated *in vitro* by poisoned food technique [20,21,22] at the concentration of 50 μg/mL. The commercial fungicides flumorph, dimethomorph, carbendazim, hymexazol and pyrimethanil were used as positive controls and they are widely used in field in China. The antifungal screening data are recorded in Table 1.

**Table 1:** The fungicidal activities of three series of pyrimidine derivatives.

Compd. No.	Fungicidal activities (50 μg/mL, inhibition rate %)													
	AK	AM	AS	BC	BM	CA	GF	GZ	MK	PI	RC	RS	SS	TC
**1a**	45.5	21.4	41.2	29.6	34.8	13.3	20	25.9	31.3	22.7	35.0	63.5	0	80.4
**1b**	18.2	0	0	7.4	17.4	0	20	14.8	31.3	0	30	61.9	0	3.9
**2a**	18.2	0	0	40.7	17.4	0	20	0	46.9	9.1	5.0	54.0	20	2.0
**2b**	31.8	21.4	5.9	37.0	17.4	13.3	26.7	29.6	46.9	0	5.0	60.3	0	2.0
**3a**	27.3	21.4	11.8	7.4	26.1	33.3	0	7.4	21.9	0	5.0	57.1	0	39.2
**3b**	63.6	50	35.3	25.9	39.1	46.7	40	44.4	25.0	36.4	25.0	66.7	0	76.5
**3c**	27.3	21.4	0	3.7	13.0	46.7	13.3	22.2	0	36.4	0	55.6	33.3	52.9
**4a**	40.9	0	0	18.5	17.4	6.7	6.7	7.4	31.3	0	20	61.9	0	19.6
**4b**	27.3	21.4	0	14.8	13.0	0	46.7	14.8	46.9	18.2	0	55.6	40	15.7
**4c**	22.7	21.4	0	33.3	26.1	6.7	53.3	40.7	37.5	13.6	0	61.9	60	0
**4d**	31.8	28.6	0	14.8	26.1	6.7	20	7.4	40.6	13.6	15.0	63.5	66.7	9.8
**Pyri.**	100	21.4	100	96.3	91.3	100	20	44.4	100	22.7	40	77.8	86.7	98.0
**Flu.**	18.2	21.4	0	7.4	17.4	0	13.3	7.4	21.9	4.5	20	61.9	0	9.8
**Dim.**	22.7	7.1	5.9	18.5	17.4	0	20	0	6.3	9.1	40	68.3	20	23.5
**Carb.**	81.8	78.6	76.5	63.0	69.6	53.3	60	59.3	43.8	27.3	100	100	73.3	98.0
**Hym.**	81.8	85.7	88.2	70.4	34.8	93.3	40	33.3	50	36.4	25.0	63.5	66.7	78.4

**Flu.** **=** flumorph, **Dim. =** dimethomorph, **Carb. =** carbendazim, **Hym. =** hymexazol, **Pyri. =** pyrimethanil.

**Figure 2:**
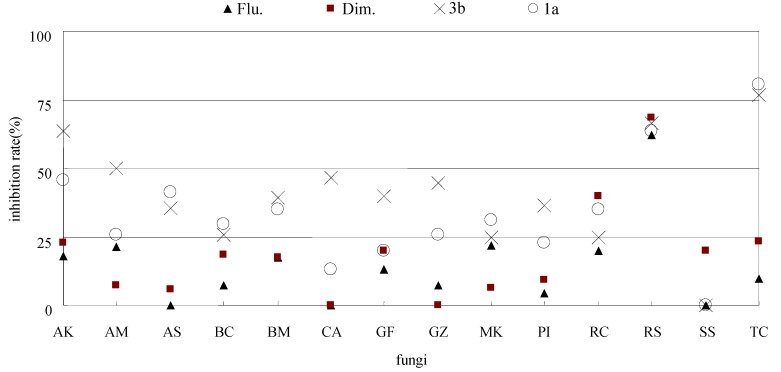
Parallel inhibition rate contrasts between positive controls (flumorph and dimethomorph) and representative pyrimidine derivatives **1a**, **3b**.

As shown in Table 1, all of the new pyrimidine derivatives exhibited certain growth inhibition effects against most of the tested fungi, and the results provided useful information to study the structure-activity relationshipx for these new structures shown in Scheme 1. The inhibition of most compounds was equal to or higher than that of the positive controls, flumorph and dimethomorph (Figure 2).

**Figure 3:**
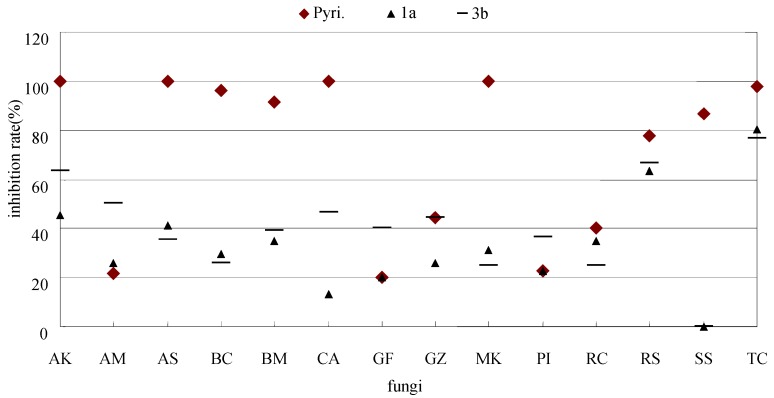
Parallel inhibition rate contrasts between leading compound pyrimethanil and **1a**, **3b**.

Especially, to PI, the activities of **1a**, **3b**, **3c**, **4b** (22.7%, 36.4%, 36.4%, 18.2%, respectively) were 1-3 times higher than dimethomorph (9.1%) which is widely used to prevent *phytophthora infestans* in filed [23,24]. Thus, the findings demonstrate that the new synthesized pyrimidine derivatives represent a new structure skeleton for inhibiting PI. The activities of carbendazim and hymexazol were excellent (>50%) to most of tested fungi, however, they showed rather lower inhibition to BM, GZ, MK and PI than several new synthesized compounds (**3b**, **3c**, **4b**, *et al*.). The leading compound pyrimethanil showed excellent effect on most of the tested fungi, except on AM, GF GZ and PI (21.4%, 20%, 44.4%, and 22.7%). It exhibited parallel activity with **3b** to GZ (44.4%), lower than **3b** and **3c** to PI (36.4% and 36.4%, respectively), at least 1-fold lower than **3b**, **4b** and **4c** to GF (40%, 46.7% and 53.3%, respectively) and 2-fold lower to AM than **3b** (50%). **1a** and **3b** provided certain broad-spectrum to the tested fungi, and showed relatively higher activities, especially to TC (80.4% and 76.5% respectively) (Figure 3).

The inhibition against the fungi except RS, GF, SS was significantly decreased when the (6-chloropyridin-3-yl)methyl of **1a** was replaced by (6-chlorothiazole-3-yl) methyl in **1b**, especially against TC, a 12-fold decrease in activity was observed, whereas no similar SAR was observed for **2a**, **2b**. Comparing pyrimethanil and compound **2** with compound **3**, preliminary SAR presumed that 2-substituted pyrimidinamine was effective to *Botrytis* while 4-substituted pyrimidinamine was more potent to *Cercospora,* and the two classes possessed a certain degree of selectivity in inhibition to different genus. Compound **3b** with a trifluoromethoxy on the 4-position of the phenyl ring was more effective than **3a** on the tested fungi, except AS, CA, PI, SS. To a certain extent, the results indicated that –OCF_3_ containing derivatives may have good activity because of their special atom size and their characteristics to form a H bond with the potential target. *S. sclerotiorum* (SS) is one of the most nonspecific, omnivorous and successful plant pathogens with an extensive host range. At least 361 species in 64 families of plants are susceptible to SS [25]. Compounds **4b**, **4c**, **4d** showed better fungicidal activities to SS (40%, 60%, 66.7%) than **4a** (0%), which showed that fungicidal activity increased smoothly with increasing density of the electron cloud on the benzene ring. The inhibition of **4c** was equal to that of pyrimethanil (66.7%), lower than carbendazim (73.3%) and hymexazole (86.7%). Encouragingly, all of the derivatives showed good activity to RS (>50%).

## 3. Experimental

### 3.1. General

All reactions using air- or moisture-sensitive reagents were conducted under an inert nitrogen atmosphere. Anhydrous solvents were distilled prior to use. THF was distilled from sodium/benzophenone. DCM and CH_3_CN were distilled from CaH_2_ and DMF was dried over P_2_O_5_. Silica (200–300 mesh) was used for column chromatography. The IR spectra were recorded on a Bruker VERTEX 70 FT-IR. ^1^H-NMR spectra were recorded on a 400 MHz spectrometer at 25 °C using TMS as an internal reference. Coupling constants (*J*) are reported in Hertz (Hz). ^1^H-NMR splitting patterns are designated as s (singlet), d (doublet), t (triplet), q (quartet), m (multiplet). Melting points of the products were determined in open capillary tubes and uncorrected. Mass spectra were recorded with a JEOL MS-D 300 mass spectrometer. Elemental analysis was performed on a Carlo-1106 model automatic instrument.

### 3.2. Procedures for the Preparation of Title Compounds ***1-4***

*4-[(6-Chloropyridin-3-yl)methoxy]-2-isopropyl-6-methylpyrimidine* (**1a**). To a solution of 6-hydroxy-2-isopropyl-4-methylpyrimidine (0.30 g, 2 mmol) and 2-chloro-5-(chloromethyl)pyridine (0.33 g, 2 mmol) in DCM (20 mL) was added NaH (0.14 g, 6 mmol) and TBAB (0.12 g, 0.37 mmol). The mixture was refluxed for 4 h. After being cooled, the mixture was poured into water (100 mL) and DCM (80 mL), the organic layer was separated and washed by water twice, then dried over anhydrous MgSO_4_, concentrated and the residue was chromatographed to give **1a** as a white solid (0.27 g, 49%), m.p. 34–37 °C. IR (film, cm^−1^): 2966, 2929, 2871, 1589, 1566, 1460, 1338, 1163, 1105, 1043, 848, 821. ^1^H-NMR (CDCl_3_) δ 1.29 (d, *J* = 5.6 Hz, 6H, 2CH_3_), 2.42 (s, 3H, pyrimidine-CH_3_), 3.07 (m, 1H, CH), 5.44 (s, 2H, OCH_2_), 6.43 (s, 1H, pyrimidine–H), 7.33 (d, *J* = 8 Hz, 1H, pyridine–H), 7.77 (d, *J* = 8 Hz, 1H, pyridine–H), 8.51 (s, 1H, pyridine–H). *m*/*z* (EI) 277 (M^+^). Anal. Calc. for C_14_H_16_ClN_3_O (277.75): C, 60.54; H, 5.81; N, 15.13; found: C, 60.61; H, 5.93; N, 15.31.

*4-[(5-Chlorothiazole-2-yl)methoxy]-2-isopropyl-6-methylpyrimidine* (**1b**). **1b** was prepared from 6-hydroxy-2-isopropyl-4-methylpyrimidine (0.30 g, 2 mmol) and 2-chloro-5-(chloromethyl)thiazole (0.34 g, 2 mmol) by the same procedure as that of **1a**. Compound **1b** was obtained as a white solid (0.22 g, 39%), m.p. 114–118 °C. IR (film, cm^−1^): 2970, 2933, 2871, 1666, 1525, 1417, 1392, 1363, 1095, 1049, 846. ^1^H-NMR (CDCl_3_) δ 1.30 (d, *J* = 8.8 Hz, 6H, 2CH_3_), 2.25 (s, 3H, pyrimidine–CH_3_), 3.17 (m, 1H, CH), 5.29 (s, 2H, OCH_2_), 6.21 (s, 1H, pyrimidine–H), 7.53 (s, 1H, thiazole–H). *m/z* (EI) 283 (M^+^). Anal. Calc. for C_12_H_14_ClN_3_OS (283.78): C, 50.79; H, 4.97; N, 14.81; found: C, 50.67; H, 4.54; N, 14.68.

*2-[(6-Chloropyridin-3-yl)methyl]amino-4,6-dimethoxypyrimidine* (**2a**). A mixture of 2-amino-4,6- dimethoxypyrimidine (0.31 g, 2 mmol), 2-chloro-5-(chloromethyl)pyridine (0.33 g, 2 mmol) and KI (0.34 g, 2 mmol) in *i*-PrOH (10 mL) was refluxed for 6–7 h, then KOH (0.12 g, 2 mmol) was added and the mixture was stirred for another 0.5 h. After being cooled to r.t., the mixture was poured into crushed ice water, the precipitate was collected and recrystallized to give **2a** as a white solid (0.39 g, 70%), m.p. 81–83 °C. IR (film, cm^−1^): 3030, 2970, 2949, 1737, 1591, 1365, 1207, 750, 663. ^1^H-NMR (CDCl_3_) δ 3.82 (s, 6H, 2OCH_3_), 4.59 (d, *J* = 6.4 Hz, 2H, CH_2_), 5.30 (m, 1H, NH), 5.45 (s, 1H, pyrimidine–H), 7.29 (d, *J* = 9.6 Hz, 1H, pyridine–H), 7.67 (dd, *J* = 2.4 Hz, 9.6 Hz, 1H, pyridine–H), 8.39 (d, *J* = 1.6 Hz, 1H, pyridine–H). *m/z* (EI) 280 (M^+^). Anal. Calc. for C_12_H_13_ClN_4_O_2_ (280.71): C, 51.34; H, 4.67; N, 19.96; found: C, 51.48; H, 4.85; N, 20.04.

*2-[(2-Chlorothiazol-5-yl)methyl]amino-4,6-dimethoxypyrimidine* (**2b**)*.* **2b** was prepared from 2-amino-4,6-dimethoxypyrimidine (0.31 g, 2 mmol) and 2-chloro-5-(chloromethyl)thiazole (0.34 g, 2 mmol) by the same procedure as that of **2a**. Compound **2b** was obtained as a white solid (0.19 g, 33%), m.p. 155–157 °C. IR (film, cm^−1^): 3242, 2970, 2947, 2349, 2337, 1737, 1591, 1365, 1215, 1051, 673, 663. ^1^H-NMR (CDCl_3_) δ 3.87 (s, 6H, 2OCH_3_), 4.69 (d, *J* = 6.4 Hz, 2H, CH_2_), 5.49 (s, 1H, pyrimidine–H), 5.56 (t, *J* = 6.4 Hz, 1H, NH), 7.43 (s, 1H, thiazole–H). *m/z* (EI) 286 (M^+^). Anal. Calc. for C_10_H_11_ClN_4_O_2_S (286.74): C, 41.89; H, 3.87; N, 19.54; found: C, 41.75; H, 3.95; N, 19.39.

*Preparation of 4-chloro-2-isopropyl-6-methylpyrimidine.* 6-hydroxy-2-isopropyl-4-methylpyrimidine (1.52 g, 10 mmol) was added to a 100mL flask, followed by addition of POCl_3_ (15 mL). The mixture was stirred at 70 °C for 2 h. The residuary POCl_3_ was distilled out and the residue was redissolved in EtOAC (200 mL). The mixture was poured into 200 mL of ice water, alkalified to pH 7 by Na_2_CO_3 _powder. The EtOAC layer was separated and washed by water twice. The organic layer was dried over anhydrous MgSO_4_ and concentrated. The obtained crude product was purified by gel silica column chromatography to give a light-yellow oil (1.55 g, 91%) which was used for the next step.

*2-Isopropyl-6-methyl-N-phenylpyrimidin-4-amine* (**3a**). A mixture of 4-chloro-2-isopropyl-6-methyl- pyrimidine (0.34 g, 2 mmol) and aniline (0.28 g, 3 mmol) in DMF (10 mL) was stirred at ~90 °C for 2 h. After the mixture was cooled to r.t., 200 mL of water was added and the mixture was extracted with EtOAC. The combined organic layer was dried over anhydrous MgSO_4_, concentrated and the residue was purified by silica gel column chromatography to give **3a** as a brown solid (0.36 g, 79%), m.p. 80–81 °C. IR (film, cm^−1^): 3286, 3172, 2927, 2869, 1581, 1510, 1498, 1442, 1363, 1247, 979, 754. ^1^H-NMR (CDCl_3_) δ 1.32 (d, *J* = 7.2 Hz, 6H, 2CH_3_), 2.34 (s, 3H, CH_3_), 3.02 (m, 1H, CH), 6.41 (s, 1H, pyrimidine–H), 7.06 (s, 1H, NH), 7.10–7.42 (m, 5H, Ph–H). *m/z* (EI) 227 (M^+^). Anal. Calc. for C_14_H_17_N_3 _(227.30): C, 73.98; H, 7.54; N, 18.49; found: C, 73.67; H, 7.62; N, 18.33.

*2-Isopropyl-6-methyl-N-(4-(trifluoromethoxy)phenyl)pyrimidin-4-amine* (**3b**). To a solution of 4-chloro-2-isopropyl-6-methylpyrimidine (0.17 g, 1 mmol) and 4-(trifluoromethoxy)aniline (0.27 g, 1.5 mmol) in *i*-PrOH (20 mL) was added anhydrous powdered K_2_CO_3_ (0.14 g, 1 mmol). The mixture was refluxed overnight. The *i*-PrOH was removed *in vacuo*, and the residue was disssolved in DCM and water. The DCM layer was seperated and the water layer was extracted with DCM, the combined organic layer was dried over anhydrous MgSO_4_, concentrated and the residue was purified by column chromatography on silica gel to give **3b** as an orange solid (0.25 g, 80%), m.p. 104–109 °C. IR (film, cm^−1^): 3296, 3198, 2970, 2931, 2873, 1587, 1508, 1415, 1247, 1201, 1163, 1016, 981, 839. ^1^H-NMR (CDCl_3_) δ 1.32 (d, *J* = 4.8 Hz, 6H, 2CH_3_), 2.37 (s, 3H, pyrimidine–CH_3_), 3.04 (m, 1H, CH), 6.35 (s, 1H, pyrimidine–H), 6.72 (s, 1H, NH), 7.47 (d, *J* = 16.4 Hz, 2H, Ph–H), 7.22 (d, *J* = 11.2 Hz, 2H, Ph–H). *m/z* (EI) 311 (M^+^). Anal. Calc. for C_15_H_16_F_3_N_3_O (311.30): C, 57.87; H, 5.18; N, 13.50; found: C, 57.74; H, 5.21; N, 13.68.

*N-cyclohexyl-2-isopropyl-6-methylpyrimidin-4-amine* (**3c**). To a solution of 4-chloro-2-isopropyl-6- methylpyrimidine (0.17 g, 1 mmol) and cyclohexane (1.0 g, 10 mmol) in CH_3_CN (10 mL) was added powdered K_2_CO_3_ (0.14 g, 1 mmol). The mixture was stirred at reflux for 7 h. After the solvent was removed in vacuo, the residue was dissolved in DCM and water, acidified by cold 0.5 N HCl aqueous to pH 7~7.5. The DCM layer was separated and the water layer was extracted with DCM for three times. The combined organic layer was dried over anhydrous MgSO_4_. The solvent was evaporated *in vacuo* to give **3c** as a light-brown solid (0.16 g, 69%), m.p. 93–97 °C. IR (film, cm^−1^): 3263, 2929, 2854, 1598, 1500, 1448, 1359, 1191, 975, 839. ^1^H-NMR (CDCl_3_) δ 1.18–1.27 (m, 10H, 2CH_3_, cyclohexane–H), 1.33–2.03 (m, 7H, cyclohexane–H), 2.33 (s, 3H, CH_3_), 2.92 (m, 1H, CH), 4.81 (m, 1H, NH), 5.97 (s, 1H, pyrimidine–H). *m/z* (EI) 233 (M^+^). Anal. Calc. for C_14_H_23_N_3_ (233.35): C, 72.06; H, 9.93; N, 18.01; found: C, 72.17; H, 9.87; N, 17.92.

*Preparation of 4-morpholinecarbonyl chloride.* Triphogene (1.49 g, 5 mmol) was dissolved in DCM (150 mL), then a solution of morpholine (0.87 g, 10 mmol) and triethylamine (1.52 g, 15 mmol) in DCM (30 mL) was added dropwise slowly in a salt-ice bath. After the reaction was completed (monitored by Iodine smoked TLC), phosgene was blown away by N_2_, then the mixture was filtered and the filtrate was concentrated to give a light-brown oil (1.41 g, 94%, with a content of about 70%) which was used for the next step without further purification.

*2-Isopropyl-6-methylpyrimidin-4-yl morpholine-4-carboxylate* (**4a**). To a stirred solution of 6-hydroxy- 2-isopropyl-4-methylprimidine (0.15 g, 1 mmol) and 4-morpholinecarbonyl chloride (crude product, 0.28 g, about 1.3 mmol) in DCM (15 mL) was added a solution of Et_3_N (0.20 g, 2 mmol) in DCM (5 mL), followed by addition of DMAP (0.03 g, 0.25 mmol). The mixture was stirred at r.t. for 5 h. 80 mL of DCM was added and the mixture was washed by cold sat. Na_2_CO_3_ aqueous and water sequentially. The DCM layer was separated and dried over anhydrous MgSO_4_, concentrated *in vacuo* to give crude product, which was purified by column chromatography on gel silica to give **4a** as a light-yellow oil (0.19 g, 72%). IR (film, cm^−1^): 2968, 2927, 2864, 1733, 1585, 1421, 1342, 1274, 1230, 1155, 1118, 1060, 848, 746. ^1^H-NMR (CDCl_3_) δ 1.32 (d, *J* = 6.8 Hz, 6H, 2CH_3_), 2.52 (s, 3H, CH_3_), 3.14 (m, 1H, CH), 3.58–3.78 (m, 8H, morpholine–H), 6.86 (s, 1H, pyrimidine–H). *m/z* (EI) 265 (M^+^). Anal. Calc. for C_13_H_19_N_3_O_3_(265.31): C, 58.85; H, 7.22; N, 15.84; found: C, 58.78; H, 7.41; N, 15.98.

*2-Isopropyl-6-methylpyrimidin-4-yl 4-fluorobenzoate* (**4b**). To a solution of 6-hydroxy-2-isopropyl- 4-methylpyrimidine (1.00 g, 6.5 mmol) in anhydrous THF (30 mL) was added the *p*-fluorobenzoyl chloride (1.43 g, 9 mmol) dropwise in an ice-water bath, followed by addition of K_2_CO_3_ powder (0.55 g, 4.0 mmol). The mixture was stirred overnight at r.t. After the THF was evaporated *in vacuo*, DCM (200 mL) was added and washed by water three times. The DCM solution was dried over MgSO_4_ and concentrated to give a crude product, which was purified by column chromatography on gel silica to give **4b** as a white solid (1.6 g, 90%), m.p. 36–37 °C. IR (film, cm^−1^): 2968, 2929, 2875, 2360, 2341, 1749, 1733, 1716, 1699, 1558, 1541, 1456, 1419, 1247, 1149, 1060,852. ^1^H-NMR (CDCl_3_) δ 1.49 (d, *J* = 9.2 Hz, 6H, 2CH_3_), 2.57 (s, 3H, CH_3_), 3.19 (m, 1H, CH), 6.94 (s, 1H, pyrimidine–H), 7.19 (t, *J* = 11.2 Hz, 2H, Ph–H), 8.24 (dd, *J* = 11.2 Hz, 7.2 Hz, 2H, Ph–H). *m/z* (EI) 274 (M^+^). Anal. Calc. for C_15_H_15_FN_2_O_2_(274.29): C, 65.68; H, 5.51; N, 10.21; found: C, 65.84; H, 5.66; N, 10.17.

*2-Isopropyl-6-methylpyrimidin-4-yl 4-chlorobenzoate* (**4c**). To a stirred solution of 6-hydroxy- 2-isopropyl-4-methylpyrimidine (0.30 g, 2 mmol), Et_3_N (0.30 g, 3 mmol) and DMAP (0.06 g, 0.5 mmol) in DCM (20 mL) was added, then a solution of *p*-chlorobenzoyl chloride (0.42 g, 2.4 mmol) in DCM was added dropwise in an ice-water bath. The mixture was warmed to r.t. and stirred for 0.5 h, then washed by water. The organic layer was dried over anhydrous MgSO_4_ and concentrated to afford a crude product, which was purified by column chromatography on gel silica to give **4c** as a colorless oil (0.41 g, 71%). IR (film, cm^−1^): 2970, 2929, 2873, 1747, 1587, 1562, 1508, 1249, 1149, 1060, 1012, 852, 757. ^1^H-NMR (CDCl_3_) δ 1.35 (d, *J* = 6.8 Hz, 6H, 2CH_3_), 2.57 (s, 3H, pyrimidine–CH_3_), 3.18 (m, 1H, CH), 7.18 (s, 1H, pyrimidine–H), 7.20 (d, 2H, *J* = 8.4 Hz, Ph–H), 8.24 (d, 2H, *J* = 8.8 Hz, Ph–H). *m/z* (EI) 290 (M^+^). Anal. Calc. for C_15_H_15_ClN_2_O_2_(290.74): C, 61.97; H, 5.20; N, 9.64; found: C, 62.06; H, 5.36; N, 9.53.

*2-Isopropyl-6-methylpyrimidin-4-yl-3,4,5-trimethoxybenzoate* (**4d**). To a solution of 3,4,5-trimethoxy-benzoicacid (0.21 g, 1 mmol) and 6-hydroxy-2-isopropyl-4-methylpyrimidine (0.15 g, 1 mmol) in DCM(10 mL) was added EDCI (0.28 g, 1.5 mmol). The mixture was stirred for 4 h. 90 mL of DCM was added and washed by water and the organic layer was separated, dried over MgSO_4_. The solvent was evaporated and the crude product was purified by chromatography on gel silica to give **4d** as a white solid (0.19 g, 55%). m.p. 97–99 °C. IR (film, cm^−1^): 2970, 2943, 2841, 1739, 1587, 1504, 1415, 1326, 1207, 1128, 974, 750. ^1^H-NMR (CDCl_3_) δ 1.35 (d, 6H, *J* = 6.8 Hz, 2CH_3_), 2.57 (s, 3H, CH_3_), 3.20(m, 1H, CH), 3.94 (s, 6H, 2OCH_3_), 3.95 (s, 3H, OCH_3_), 6.91 (s, 1H, pyrimidine–H), 7.45 (s, 2H, Ph–H). *m/z* (EI) 346 (M^+^). Anal. Calc. for C_18_H_22_N_2_O_5_ (346.38): C, 62.42; H, 6.40; N, 8.09; found: C, 62.63; H, 6.61; N, 7.98.

## 4. Conclusions

Eleven novel pyrimidine derivatives of three classes had been synthesized and identified. The bioactivity tests showed that most of them had antifungal activities. The antifungal activities of most of compounds were equal to or higher than those of the commercial fungicides flumorph and dimethomorph. Compounds **3b**, **4b** and **4c** showed better activity than the lead compound, pyrimethanil to GF. Compound **3b** was more potent to AM than pyrimethanil. Compounds **1a** and **3b** displayed certain broad-spectrum activity towards the tested fungi. Compounds **4a**, **4b**, **4c** and **4d** showed relatively moderate antifungal activity. The preliminary SAR can offer great help to design more active compounds as potent fungicides.

## Supplementary Materials

Supplementary File 1
