# Supplementary figures and images for: Synthesis and Antifungal Activities of Some Novel Pyrimidine Derivatives
Source: Molecules. 2011 Jun 30;16(7):5618–28. doi: 10.3390/molecules16075618 (PMC6264579; doi:10.3390/molecules16075618)

**IR:**

**1a**

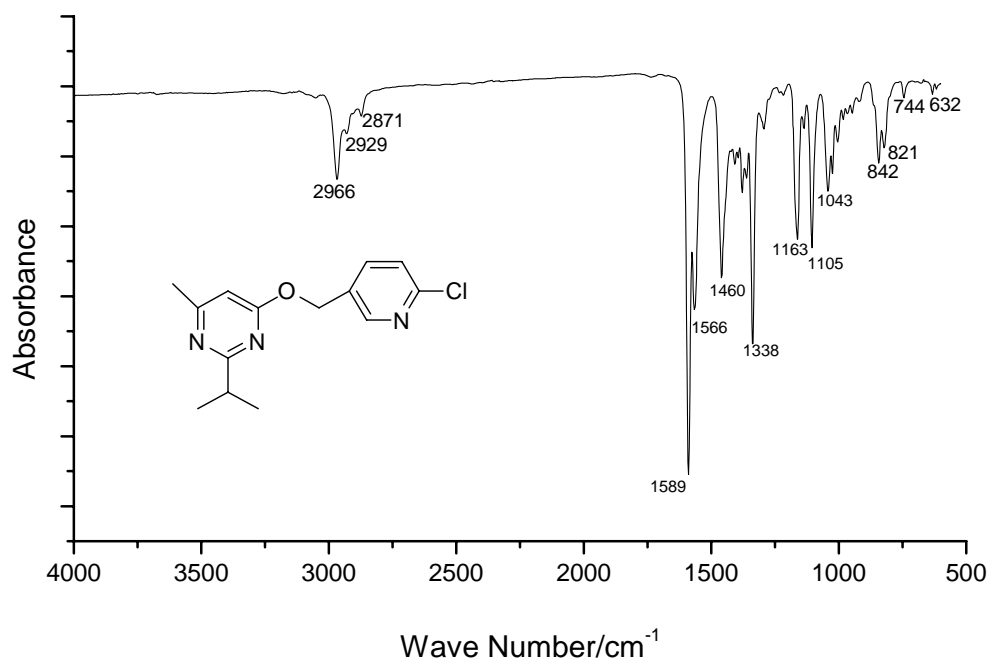

**1b**

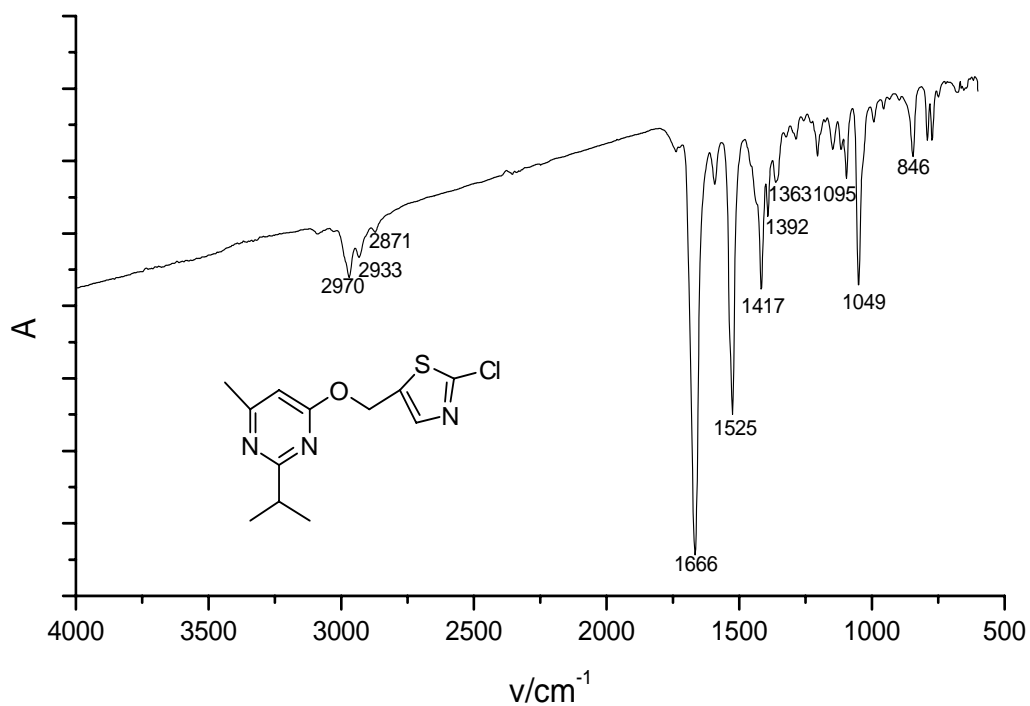

2a

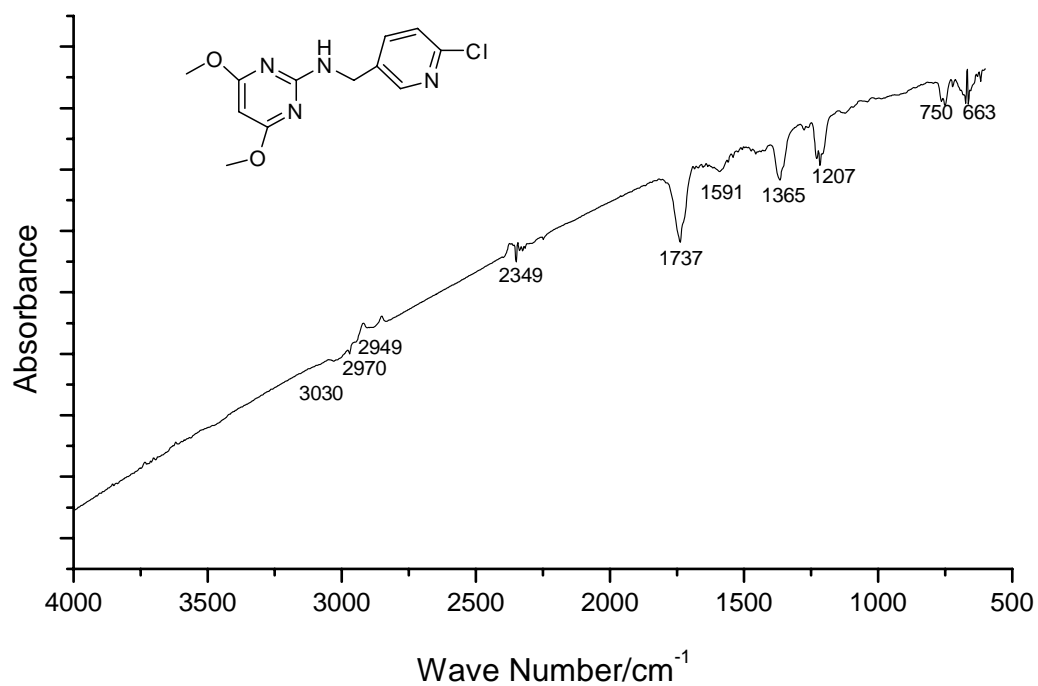

2b

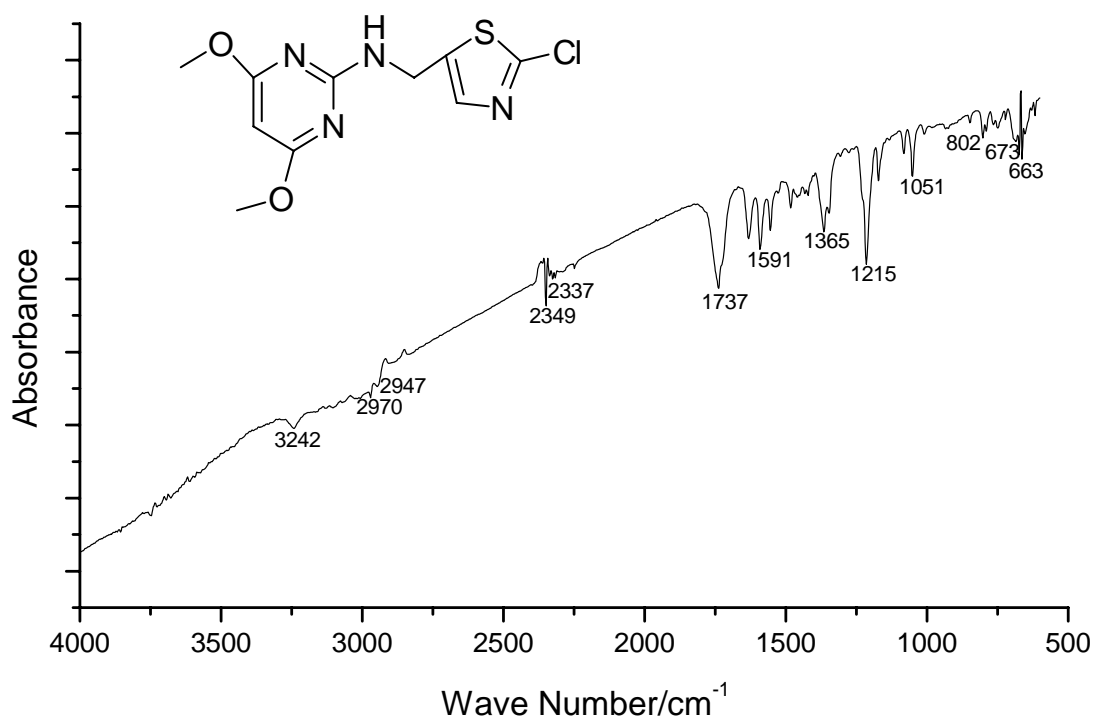

**3a**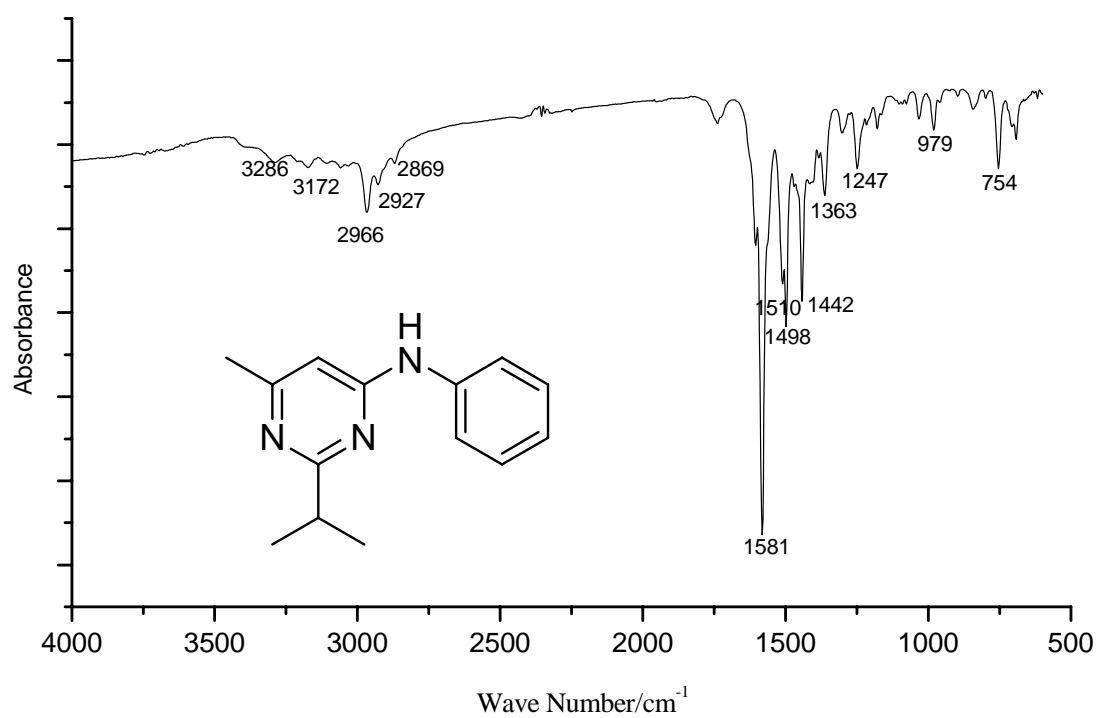**3b**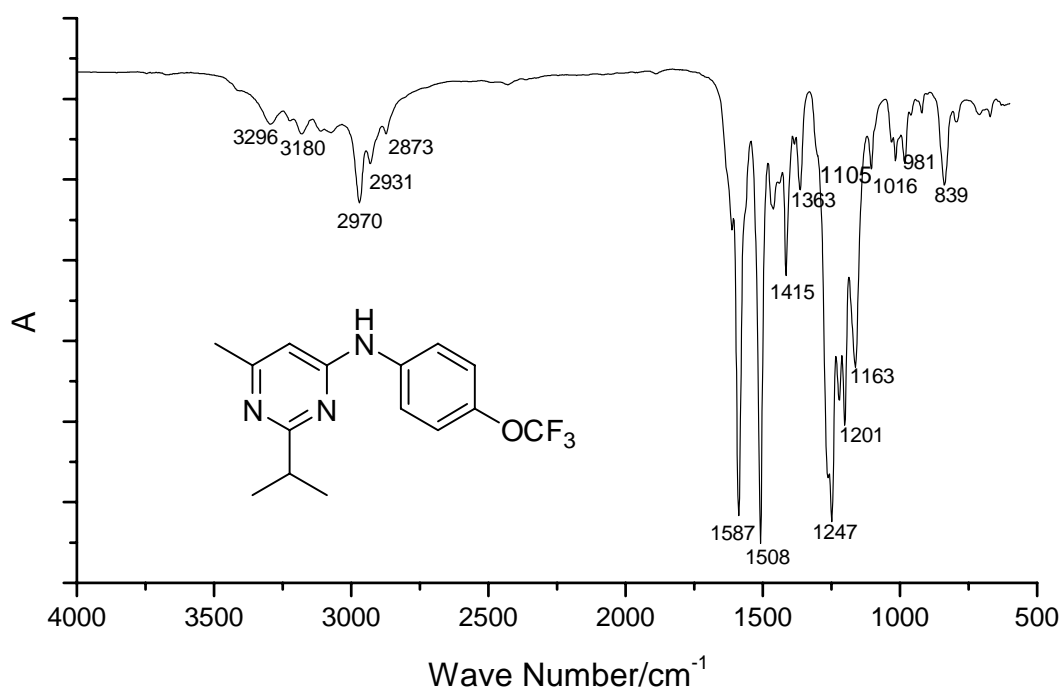

3c

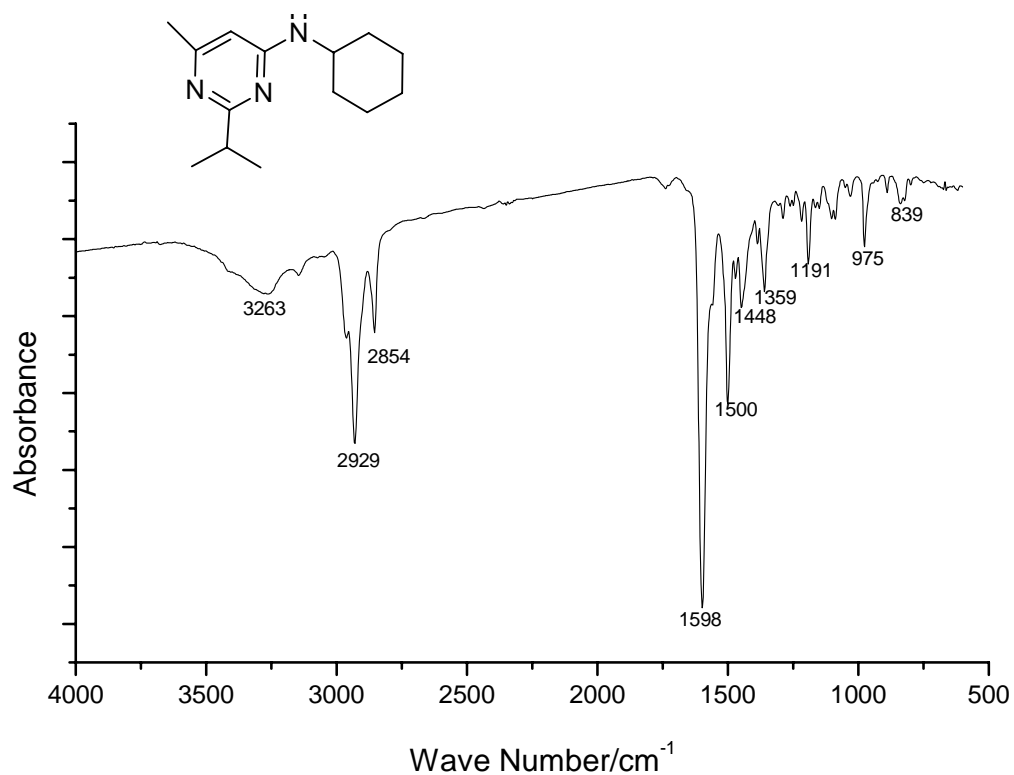

4a

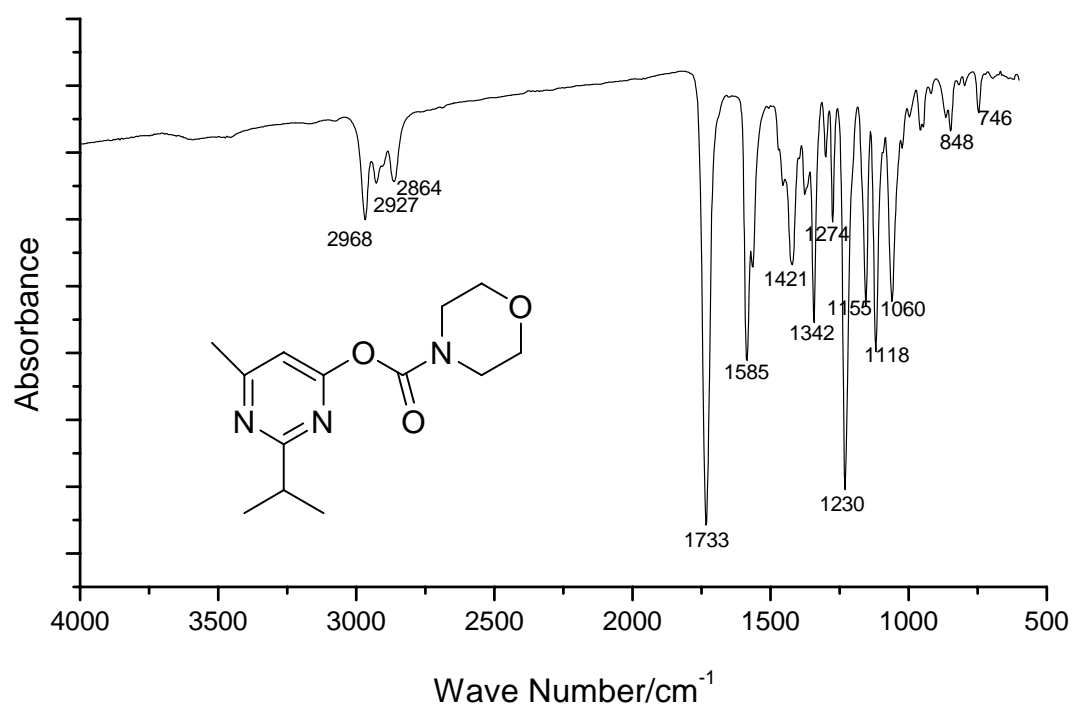

4b

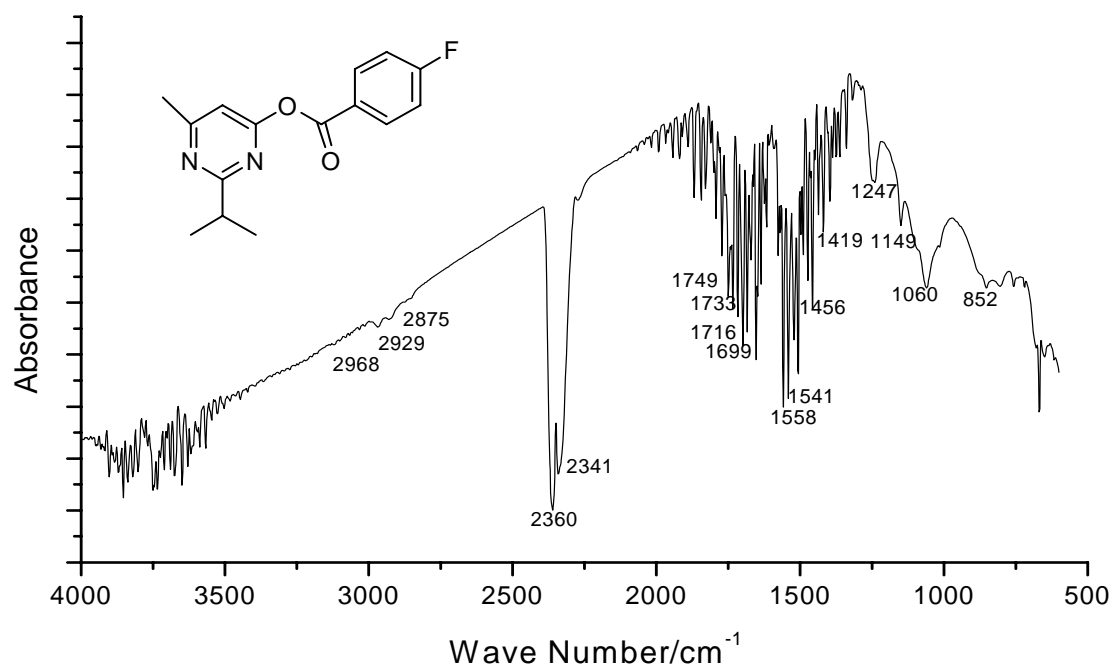

4c

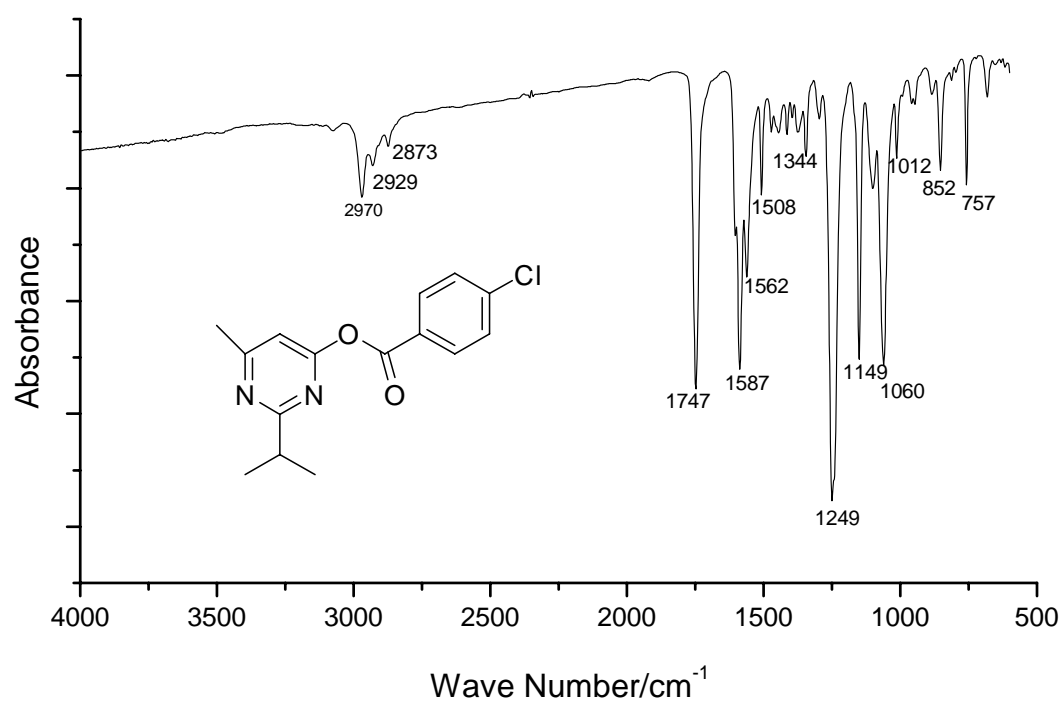

4d

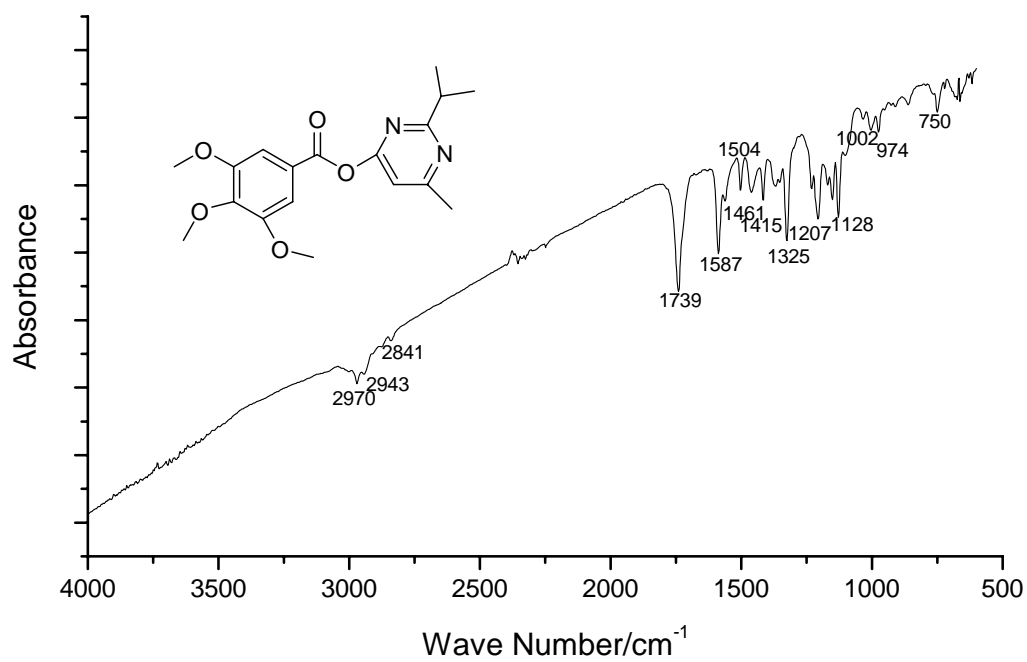

<sup>1</sup>H-NMR:

1a

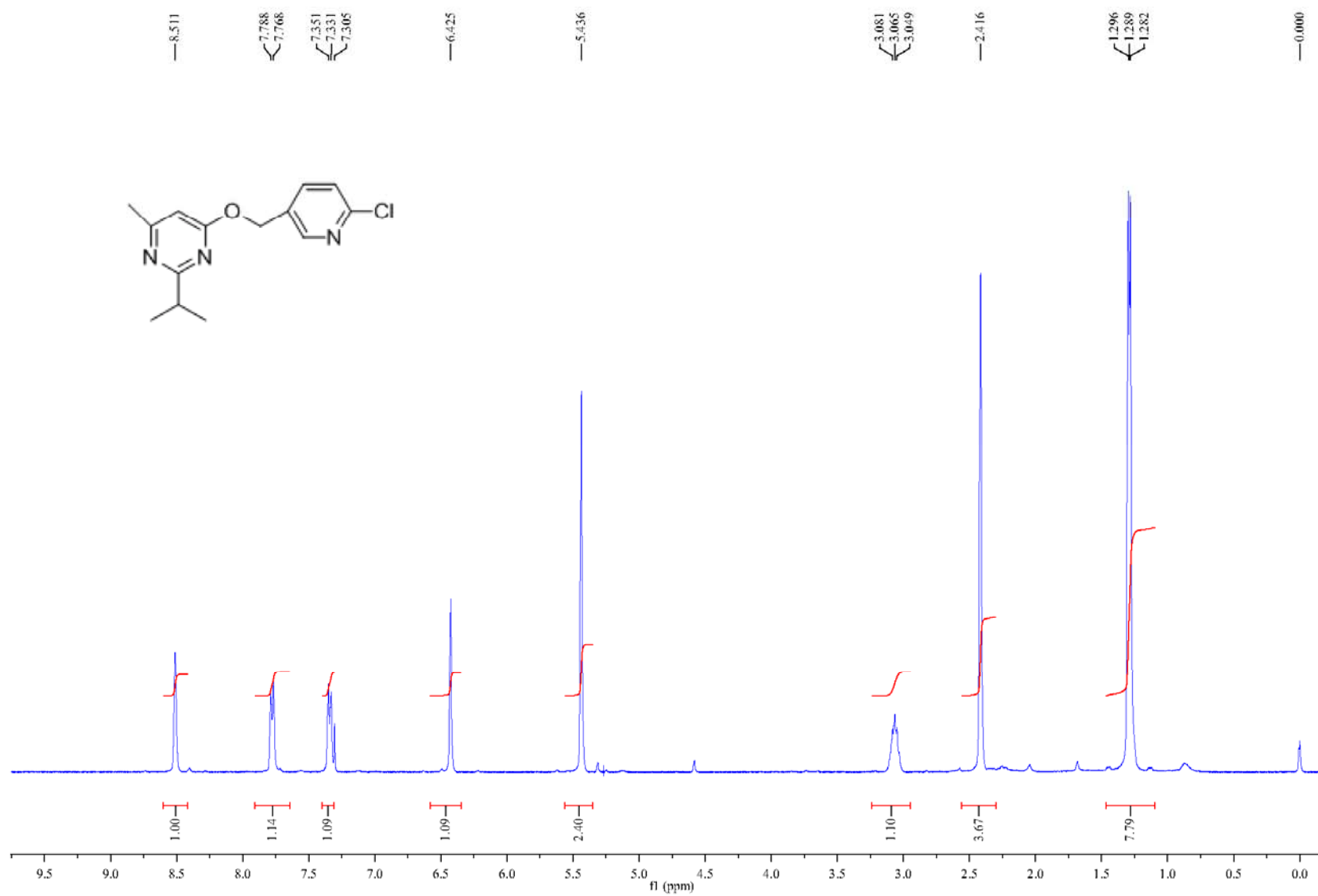

1b

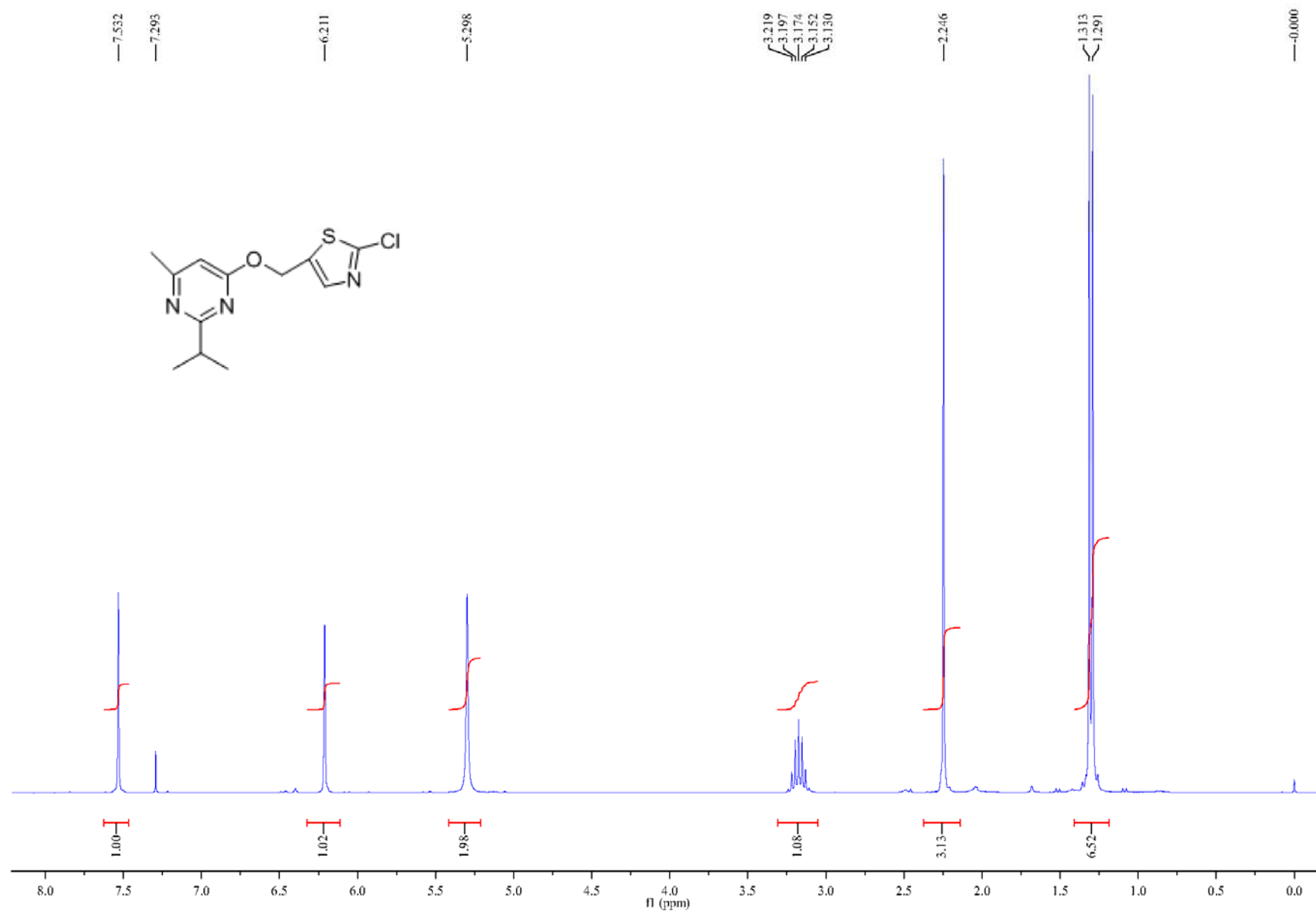

2a

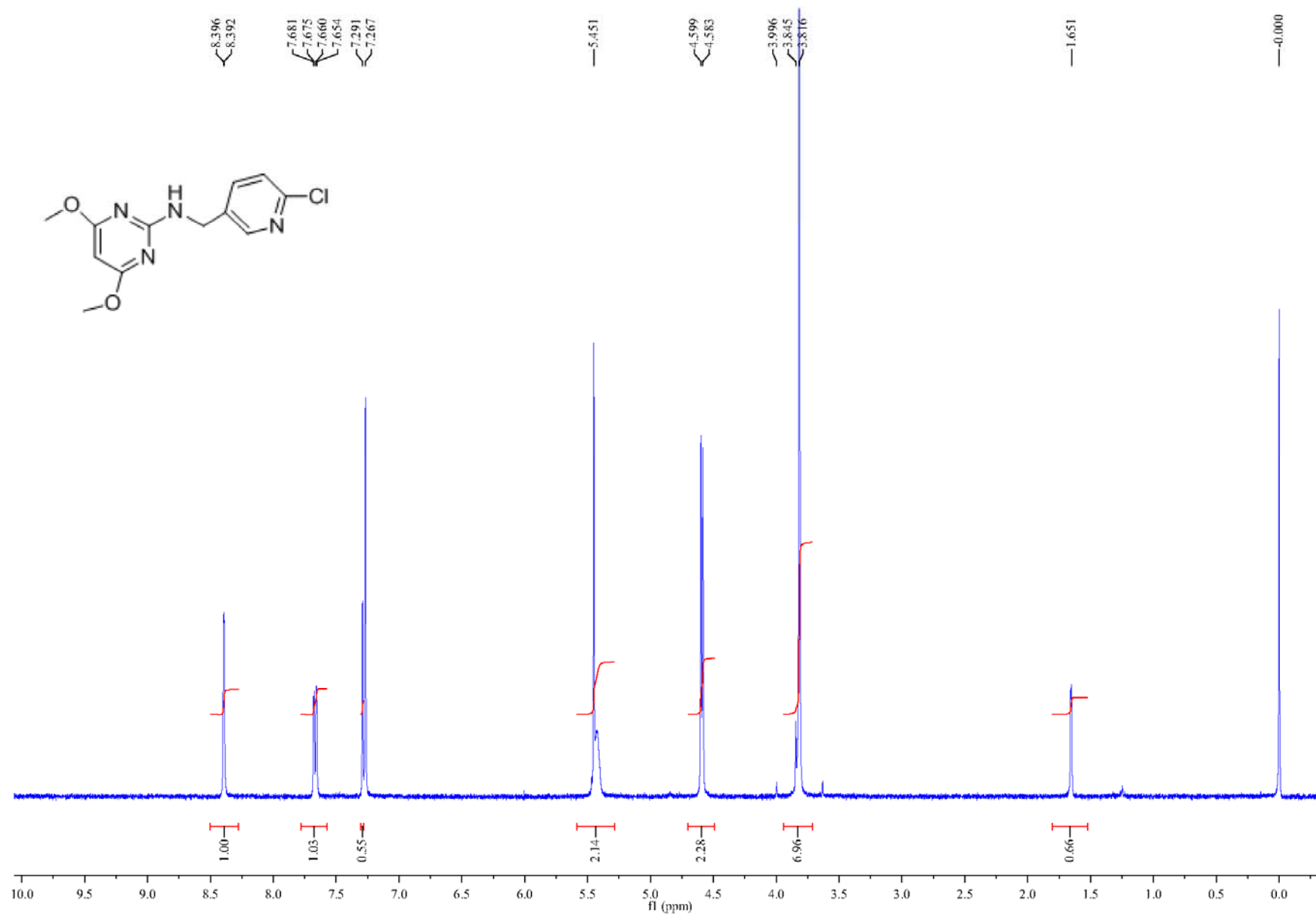

2b

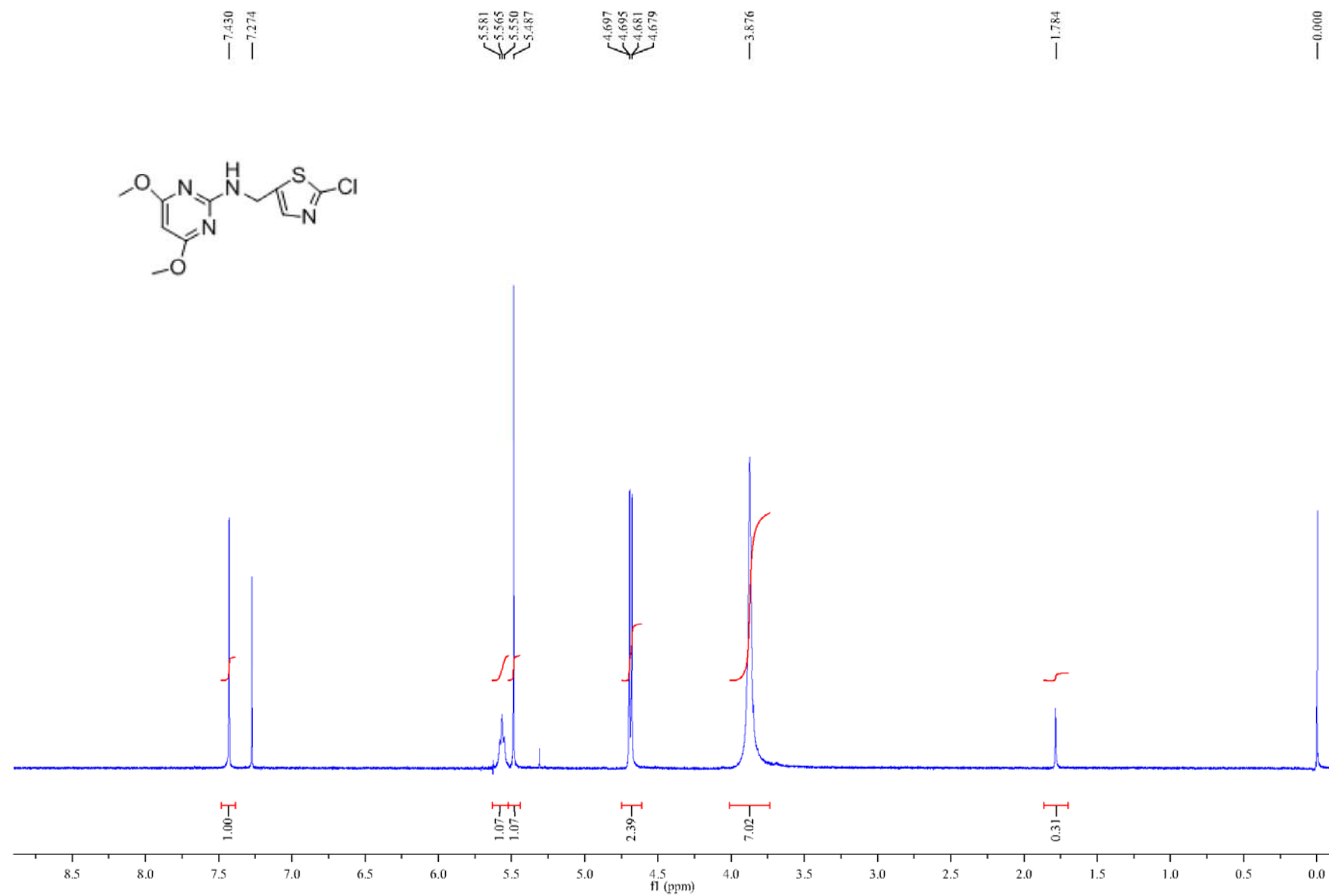

3a

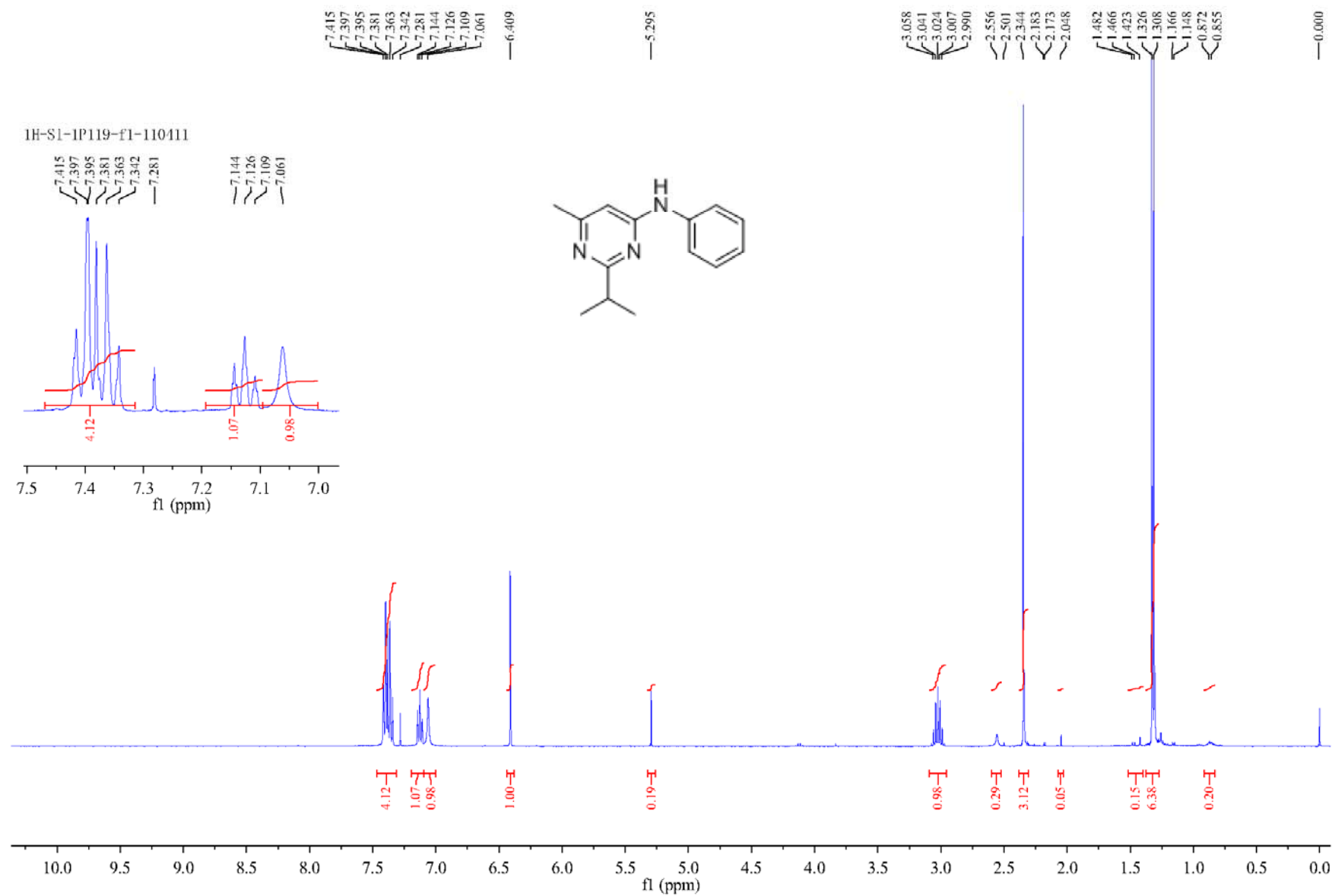

3b

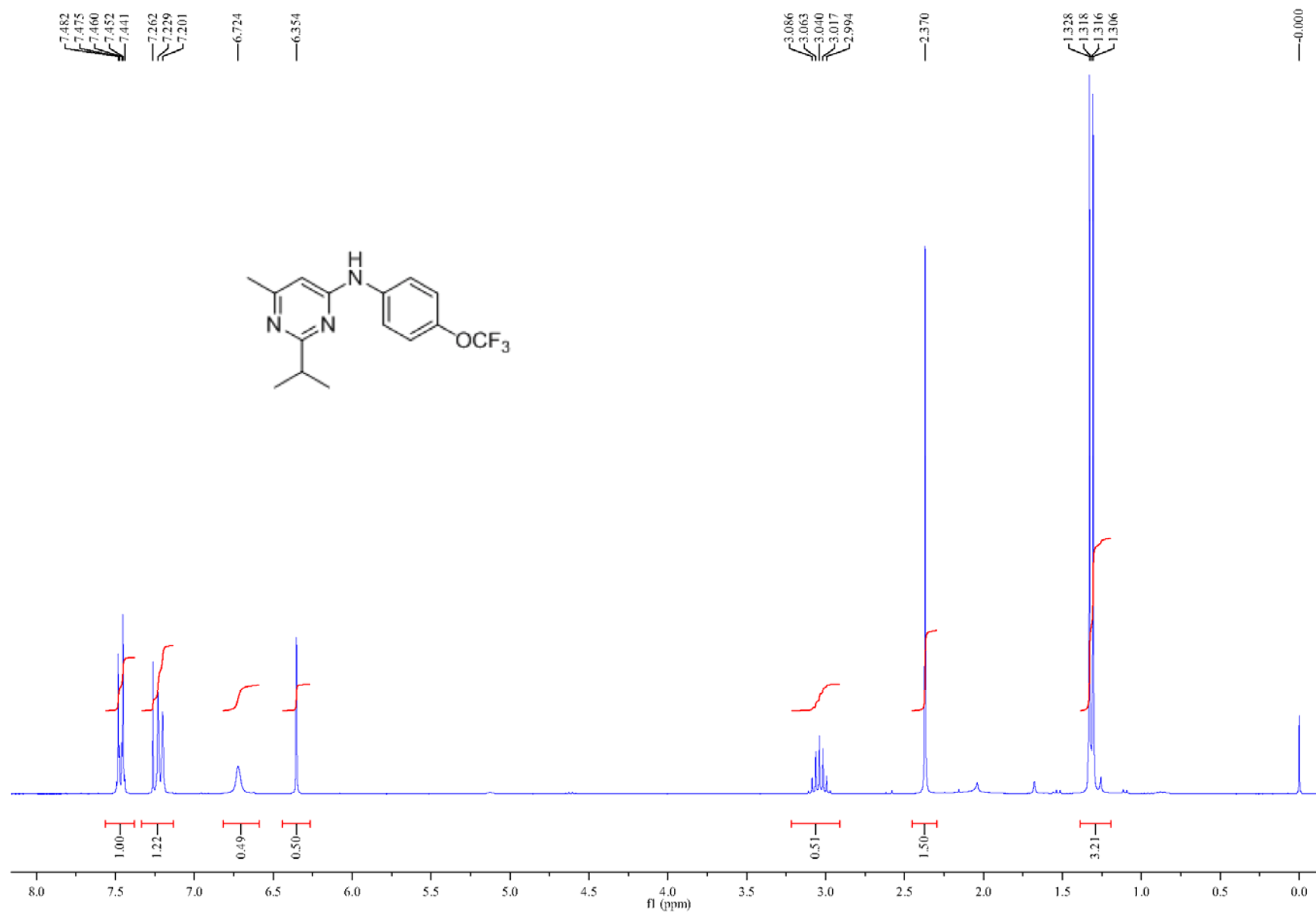

3c

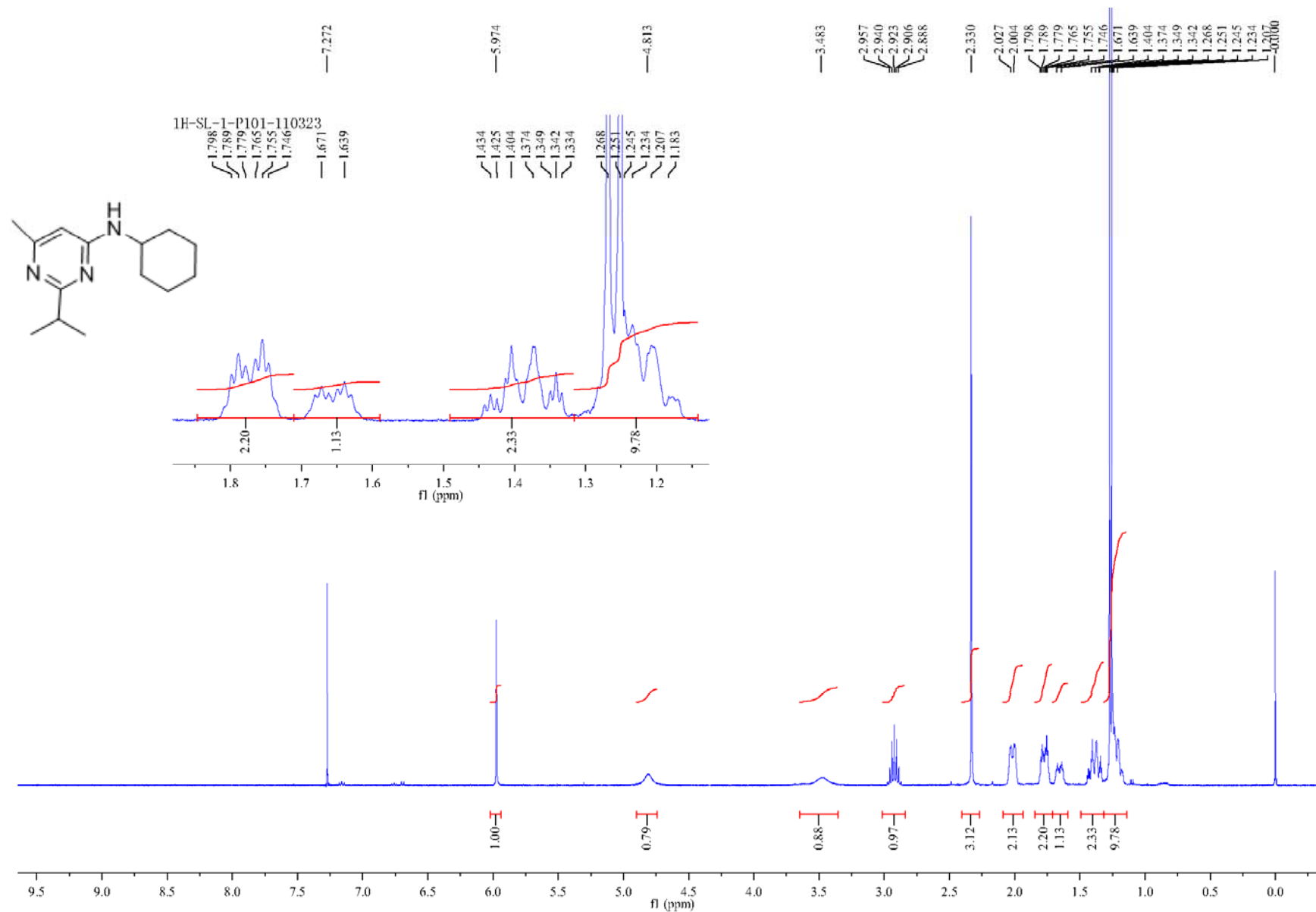

4a

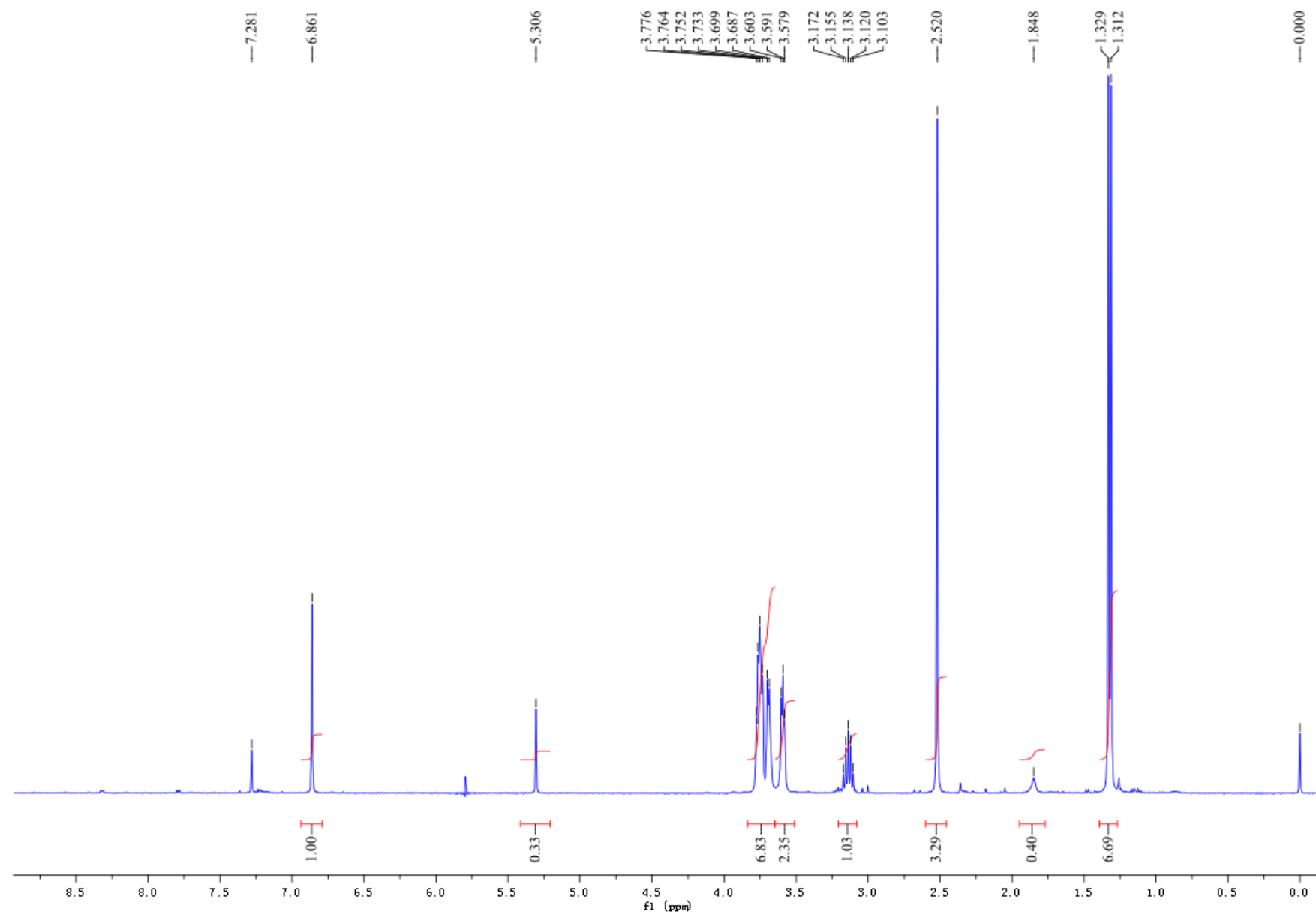

4b

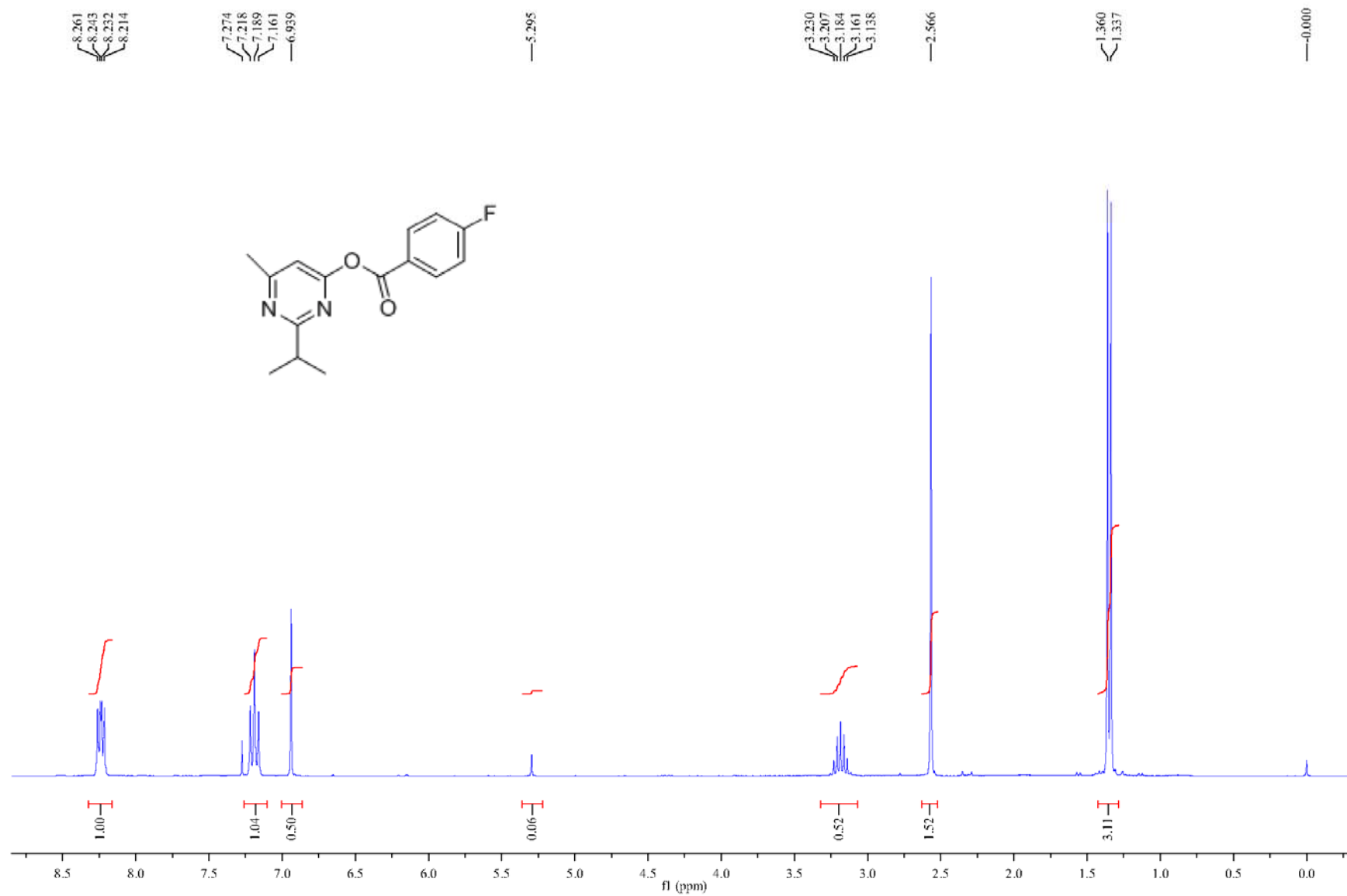

4c

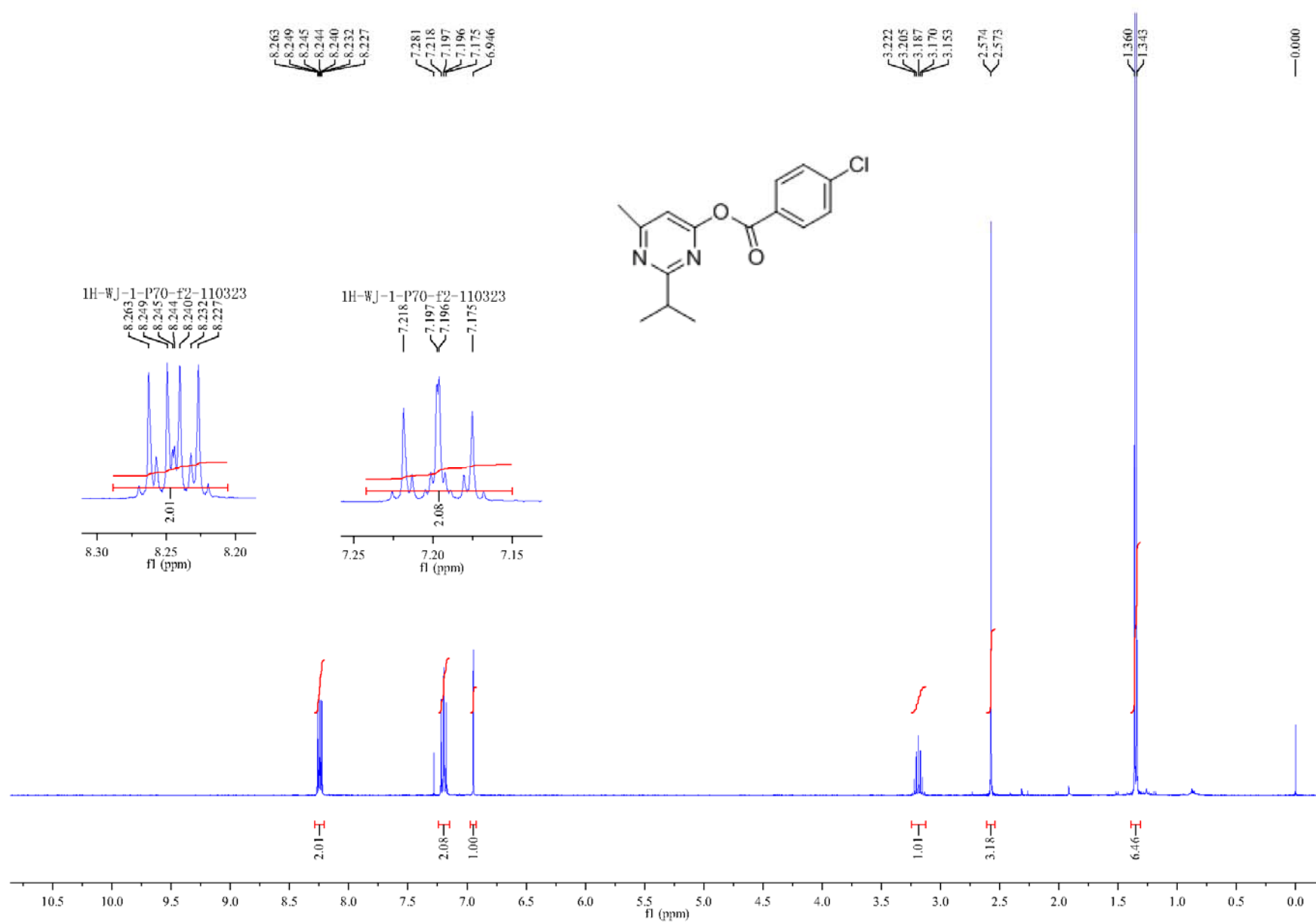

4d

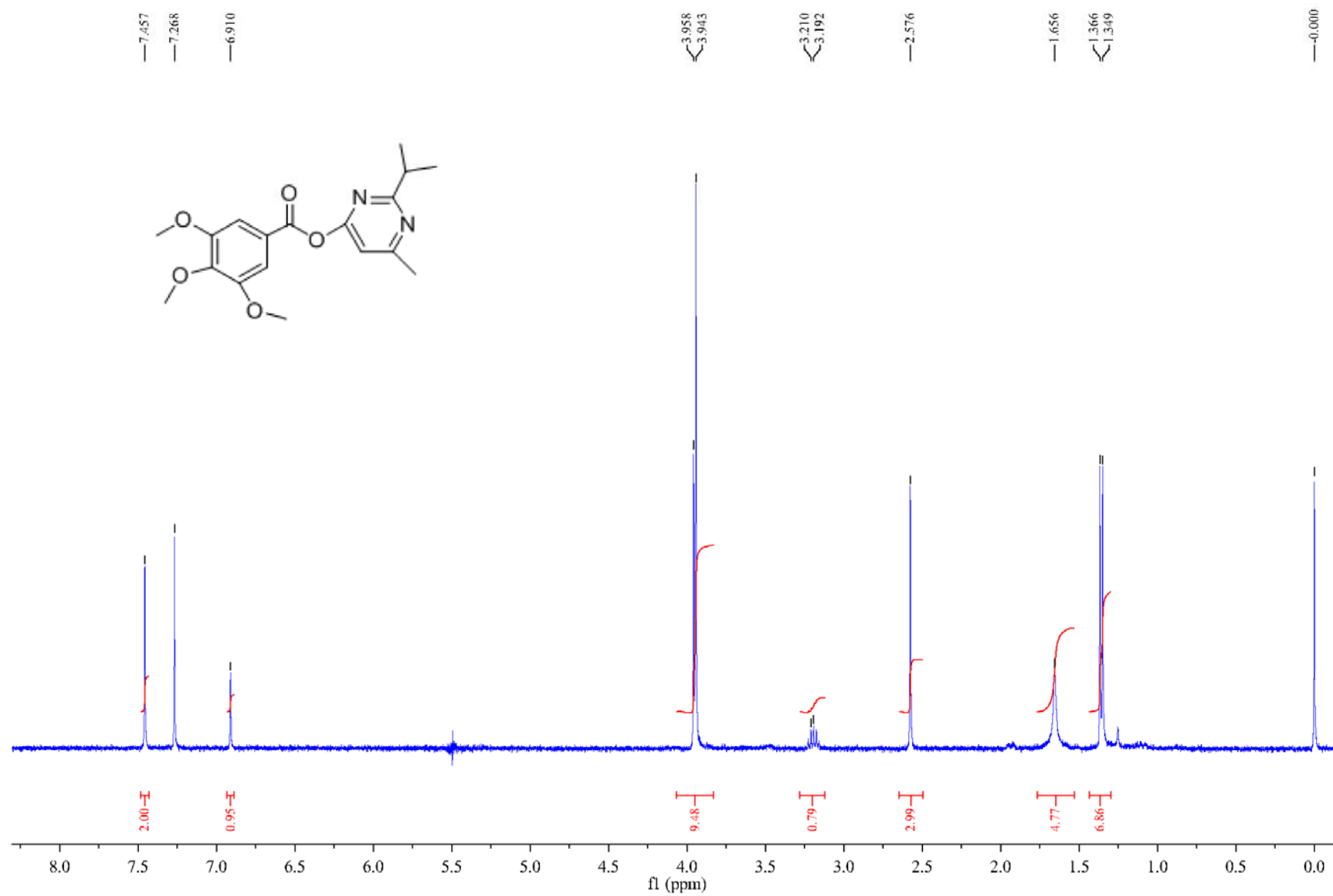

Supplement: Supplementary File 1 [file molecules-16-05618-s001.pdf]
